# Dentiradicibacter hellwigii gen. nov., sp. nov., isolated from a secondary infected root canal in the human oral cavity

**DOI:** 10.1099/ijsem.0.006690

**Published:** 2025-03-05

**Authors:** Sibylle Bartsch, Annette Wittmer, Ann-Kathrin Weber, Meina Neumann-Schaal, Jacqueline Wolf, Sabine Gronow, Jake David Turnbull, Christian Tennert, Georg Häcker, Fabian Cieplik, Ali Al-Ahmad

**Affiliations:** 1 Center for Dental Medicine, Department of Operative Dentistry and Periodontology, Faculty of Medicine and Medical Center, University of Freiburg, Freiburg, Germany; 2 Institute of Microbiology and Hygiene, Faculty of Medicine and Medical Center, University of Freiburg, Freiburg, Germany; 3 Leibniz Institute DSMZ-German Collection of Microorganisms and Cell Cultures, Braunschweig, Germany; 4 Culture Collections, UK Health Security Agency, 61 Colindale Avenue, London, NW9 5EQ, UK; 5 Department of Restorative, Preventive and Pediatric Dentistry, University of Bern, Bern, Switzerland

**Keywords:** *Betaproteobacteria*, *Dentiradicibacter*, oral cavity, root canal

## Abstract

A motile, rod-shaped and anaerobic strain WK13^T^ was isolated from a secondary root canal infection of a human tooth. WK13^T^ cells were Gram-stain-negative, catalase-positive and oxidase-negative. The major fatty acids (≥ 5.0%) were C_16 : 0_, C_18 : 0_, C_16 : 1_* ω*7c, C_18 : 1_* ω*9c and C_18 : 2_* ω*6,9c. The DNA G+C content was 57.94 mol%. The major polar lipids were phosphatidylethanolamine, phosphatidylserine, diphosphatidylglycerol, phosphatidylcholine and lysophosphatidylcholine. There were no respiratory quinones detectable. Phylogenetic analysis based on 16S rRNA gene sequences indicated that strain WK13^T^ belongs to the class *Betaproteobacteria*. WK13^T^ showed a 93.6% and 93.5% 16S rRNA gene sequence similarity to the most closely related cultured species, *Propionivibrio pelophilus* strain DSM 12018^T^ and *Propionivibrio dicarboxylicus* strain DSM 5885^T^, respectively. On the basis of physiological and biochemical data, the isolate is considered to represent a novel species of a new genus in the class *Betaproteobacteri*a, for which we propose the name *Dentiradicibacter hellwigii* gen. nov., sp. nov. The type strain is WK13^T^ (=DSM 112713^T^=NCTC 14938^T^).

## Introduction

In the human oral cavity, a total of 774 oral bacterial species have been identified to date. With regard to the expanded Human Oral Microbiome Database V3.1, 58% of these species are cultivated, and their names have been validly published, 16% are unnamed but have been cultivated and 26% are uncultivated [1,2]. Here, we present a novel bacterial taxon isolated from a tooth with a secondary endodontic infection. Endodontic infections are generally semi-specific and consist of a diverse community of micro-organisms with anaerobic dominance [3]. They occur as primary endodontic infections due to necrotic dental pulp or as secondary endodontic infections following previous endodontic treatment. Endodontic infections can spread to the periradicular tissues and lead to apical periodontitis, and secondary endodontic infections are considered the major cause of endodontic treatment failure in dentistry [4]. In addition, the possible associations of endodontic infections with certain general diseases, such as cardiovascular diseases, are the subject of current research [5]. The isolation of the bacteria involved in these processes is therefore crucial to understand their role in these infection processes and for effective treatment strategies and prevention of secondary endodontic infections. The most closely related strains to our isolated strain WK13^T^ included clone HE18 (DQ087192), clone ncd2660c12c1 (JF231593), clone 10B839 (FJ976321) and clone 071071_233 (JQ476837), which had all been identified in human samples, but have not been isolated so far [6,8]. More distantly related strains have been detected in the oral cavity of cats and dogs. These are, for example, clone PW021 (JN713386) or strain 7232 (KM461972), none of which have an isolated representative so far [9,10]. The most closely related cultured species are *Propionivibrio pelophilus* DSM 12018^T^ (formerly *Propionibacter pelophilus*) and *Propionivibrio dicarboxylicus* DSM 5885^T^, both isolated from anoxic mud [11,12]. The novel strain WK13^T^ can be distinguished from these organisms by a multitude of taxonomic parameters, which collectively allow us to propose a new genus, *Dentiradicibacter*, containing the new species *Dentiradicibacter hellwigii* for which WK13^T^ is the designated type strain.

## Isolation and ecology

Strain WK13^T^ was isolated from a female patient at the Department of Operative Dentistry and Periodontology, Center for Dental Medicine of the University Medical Center Freiburg, Germany. The patient had two teeth with secondary endodontic infections. Prior to sample collection, a rubber dam was applied, and the tooth was disinfected with 30% hydrogen peroxide and 3% sodium hypochlorite. Sodium hypochlorite was then neutralized with 5% sodium thiosulphate. After removal of the root canal fillings, 0.9% sodium chloride solution was added to each canal, and the samples were then collected using sterile paper points each. WK13^T^ was isolated from the root canal of the upper left maxillary premolar (tooth 24). The root canal samples obtained were immediately placed in reduced transport fluid (containing 25% glucose [13]) and stored at −80 °C. Samples were thawed in a 36 °C water bath. A serial dilution was done and plated on yeast extract cysteine agar with 10% sheep blood (Beerens Formulation). The medium was as follows (l^−1^): 18 g Bacto Agar, 5 g NaCl, 10 g peptone from casein, 2 g glucose, 5 g yeast extract, 2 g meat extract and 10 ml l-cysteine solution (3%); add ~6 ml KOH (10%) to adjust to pH 7; add dH_2_O to a final volume of 1.000 ml. All components were autoclaved and supplemented with 0.1 ml vitamin K1 solution (1% in ethanol), 10 ml hemin solution (0.05%) and 100 ml sheep blood directly before pouring the plates. The inoculated plates were incubated for 10 days at 37 °C under anoxic conditions (<0.1 % O_2_, 15%–20% CO_2_) with the GENbox anaer Generators (bioMérieux, Marcy l'Etoile, France) in anaerobic jars (2,5 L, Merck KGaA, Darmstadt, Germany). After the initial isolation, we were able to cultivate the strain under anoxic conditions (<0.1% O_2_, 15%–20% CO_2_) on yeast extract cysteine agar with 10% sheep blood (Beerens Formulation) in co-culture with *Capnocytophaga sputigena* A20-6 (lab isolate – available on request), *Capnocytophaga granulosa* WK-13c (NCTC 15079) or *Prevotella intermedia* DSM 20706^T^ (spread in the outer edge of the agar plates). Good growth could also be obtained with fastidious anaerobe agar with 7% defibrinated horse blood (PP1560; E and O Laboratories Ltd, Scotland, UK) or on Columbia Blood Agar (PB5039A; Thermo Scientific) with *C. sputigena*, *C. granulosa* or *P. intermedia*. The strain prefers to grow in co-cultures but can also be cultivated as a single isolate on the above-mentioned media. However, the growth is extremely poor.

For long-term storage, the strain was stored in basal glucose phosphate (BGP) medium [14] with 15% glycerol at −80 °C. The BGP medium consists of the following (l^−1^): 10 g tryptone, 5 g NaCl, 3 g beef extract, 5 g yeast extract, 0.4 g cysteine hydrochloride, 1 g glucose, 4 g Na_2_HPO_4_ and 150 ml glycerol; add dH_2_O to a final volume of 1000 ml.

## 16S rRNA gene sequence phylogenetic analysis

Strain identification was done using NCBI Nucleotide blast (https://blast.ncbi.nlm.nih.gov/Blast.cgi), and most closely related organisms were additionally detected using EZBioCloud [15]. The 16S rRNA gene sequences for the construction of the phylogenetic tree were obtained from NCBI (https://www.ncbi.nlm.nih.gov/). The phylogenetic tree was designed using mega6 [16]. The sequences were aligned by the muscle algorithm. The aligned sequences were trimmed to a common length of 1342 bp for all included sequences. Phylogenetic trees were constructed by the methods of neighbour-joining (NJ) with the Jukes–Cantor model and maximum likelihood (ML) with the Tamura–Nei model, both with 1000 bootstrap replications. *Haemophilus influenzae* DSM 11970 was used as an outgroup. Sequence similarities were calculated using ‘EMBOSS needle’ pairwise sequence alignment (PSA) with the trimmed sequences [17].

**Fig. 1.**
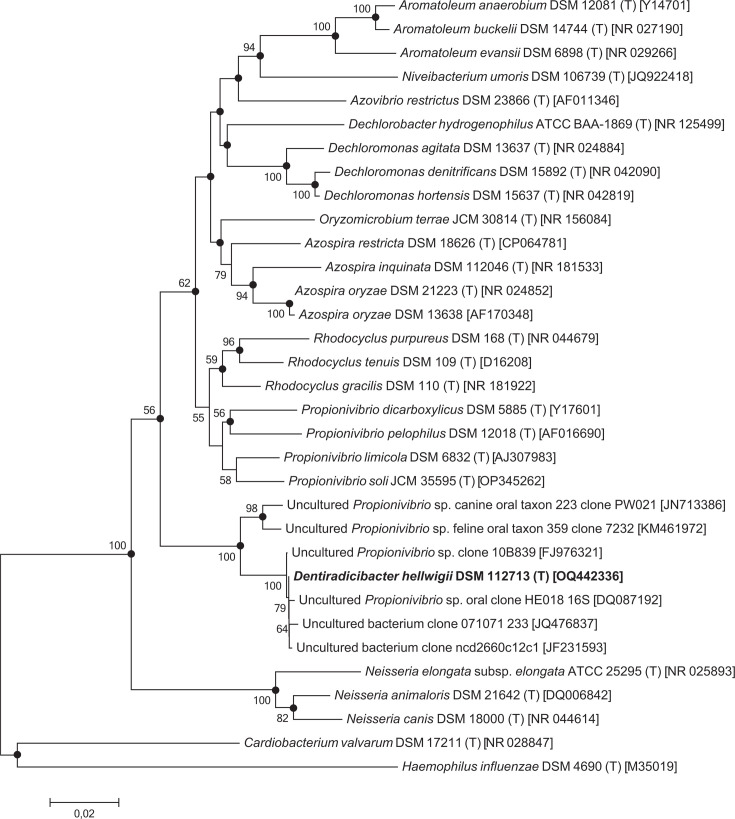
Phylogenetic analysis of 16S rRNA gene sequences showing the positions of strain WK13^T^ and closely related taxa. Bootstrap values (≥50) are expressed as percentages of 1000 replications and are shown at the branch nodes. The tree was calculated using the NJ method. Black circles in branches indicate that the corresponding nodes were also recovered using the ML method. The bar shows 0.02 substitutions per nucleotide.

Phylogenetic tree analysis showed that strain WK13^T^ represents a phylogenetic subgroup of the *Betaproteobacteria*. Strain WK13^T^ shares 16S rRNA gene sequence similarity with *Propionivibrio pelophilus* DSM 12018^T^ (93.6%), *Propionivibrio dicarboxylicus* DSM 5885^T^ (93.5%), *Propionivibrio soli* JCM 35595^T^ (92.9%), *Propionivibrio limicola* DSM 6832^T^ (93.0%), *Azospira restricta* DSM 18626^T^ (91.7%), *Rhodocyclus gracilis* DSM 110^T^ (93.1%) and *Dechloromonas hortensis* DSM 15637^T^ (90.6%) (Fig. 1). All species were isolated from environmental habitats [11,12, 18,23]. The highest 16S rRNA sequence similarities are with so far uncultured representatives isolated from human specimens: *Propionivibrio* sp. clone 10B839 from oral mucosa (99.9%), uncultured bacterium clone ncd2660c12c1 from human skin (99.9%), *Propionivibrio* sp. oral clone HE018 from subgingival plaque (99.8%) and uncultured bacterium clone 071071 233 from the human mouth (99.8%) [6,8]. These values are above the commonly used threshold for species delineation of 97% and belong presumably to the same species [24]. It should be noted, however, that these values are merely indicative and should not be employed for the purpose of rigorous validation. A bootstrap value of 100% supports this branching (Fig. 1).

## Genome sequencing and analysis

The genome was sequenced and assembled by the Deutsche Sammlung von Mikroorganismen und Zellkulturen (Braunschweig, Germany) using the PacBio sequencing technology and SMRT Link analysis software. The total assembled genome length for isolate WK13^T^ was 2 720 159 bp in three non-circular contigs. The genome was found to contain no detectable plasmids, as determined using MOB-SUITE version 3.1.8 [25]. Two rRNA gene operons were identified within the contigs using barrnap [26]. The three contigs of strain WK13^T^ are available under the accession number PRJNA1108070.

**Fig. 2.**
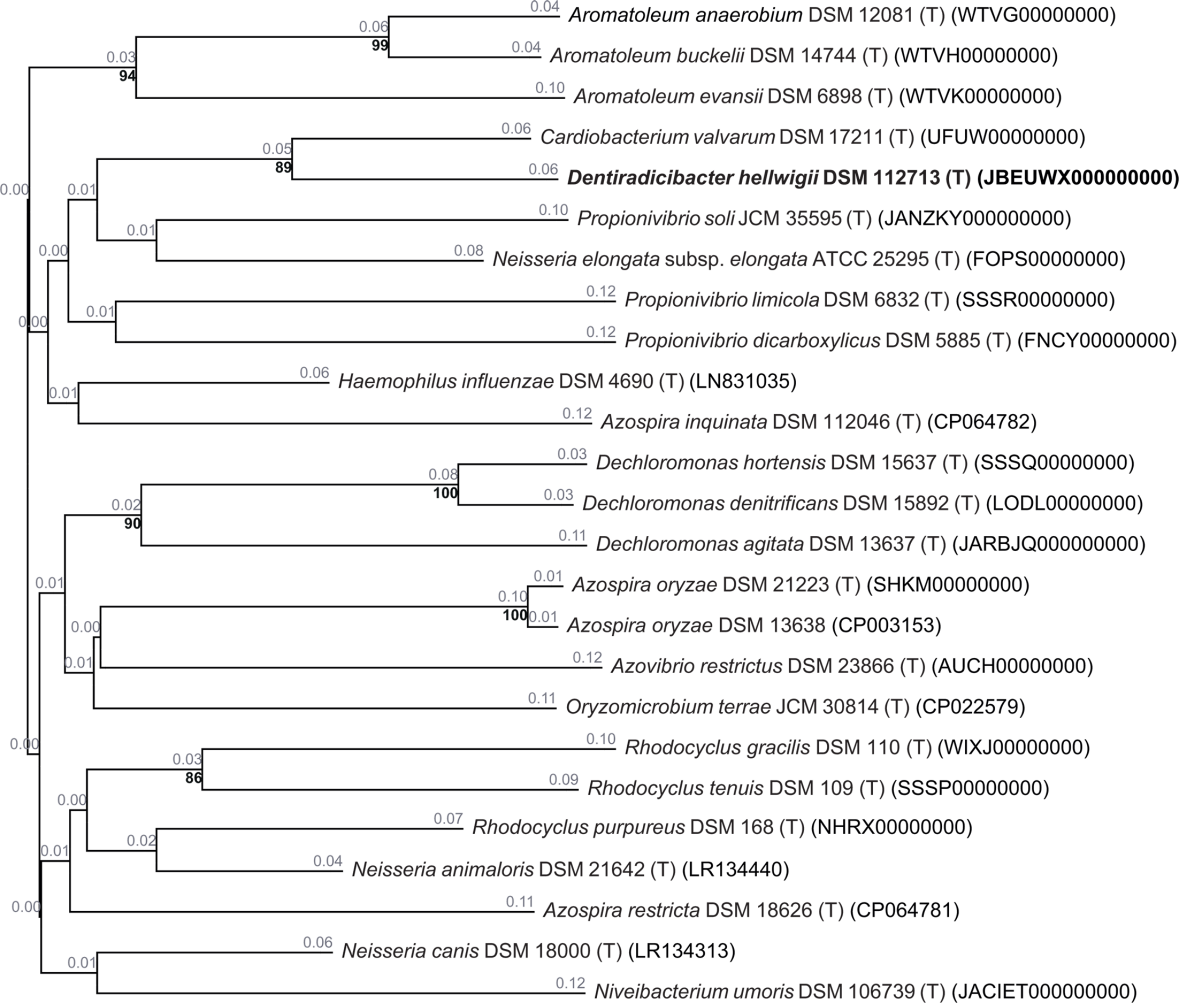
GBDP tree (whole-genome sequence-based) of strain WK13^T^ and closely related taxa. Tree inferred with FastME 2.1.6.1 from GBDP distances calculated from genome sequences [31]. The branch lengths are scaled in terms of the GBDP distance formula d5. The black bold numbers below branches are GBDP pseudo-bootstrap support values >60% from 100 replications, with an average branch support of 44.4%. The numbers in grey above the branches represent branch length values. The tree was rooted at the midpoint [32].

Sequence data were uploaded to the Type (Strain) Genome Server (TYGS), a free bioinformatics platform available under https://tygs.dsmz.de, for a whole genome-based taxonomic analysis and calculation of the G+C content [27]. The analysis also made use of recently introduced methodological updates and features [28]. Information on nomenclature, synonymy and associated taxonomic literature was provided by TYGS’s sister database, the List of Prokaryotic names with Standing in Nomenclature (available at https://lpsn.dsmz.de) [28]. The results were provided by the TYGS on 10 December 2024. In the first step, only the genome from WK13^T^ was uploaded, and TYGS determined closely related type strain genomes via the Mash algorithm, a fast approximation of intergenomic relatedness [29]. In the next step, the accession numbers for the strains of interest were uploaded to gain the same strains in the 16S rRNA gene tree (Fig. 1) and the Genome blast Distance Phylogeny (GBDP) tree (Fig. 2). Unfortunately, *P. pelophilus* has no genome accession number on NCBI and could not be included in the GBDP tree. For the phylogenomic inference, all pairwise comparisons among the set of genomes were conducted using GBDP, and accurate intergenomic distances were inferred under the algorithm 'trimming' and distance formula d5 [30]. One hundred distance replicates were calculated each. Digital DNA–DNA hybridization (dDDH) values and CIs were calculated using the recommended settings of the genome-to-genome distance calculator (GGDC) 4.0 [28,30]. The resulting intergenomic distances were used to infer a balanced minimum evolution tree with branch support via FastME 2.1.6.1 including subtree pruning and regrafting postprocessing [31]. Branch support was inferred from 100 pseudo-bootstrap replicates each. The trees were rooted at the midpoint and visualized with PhyD3 [32,33].

In the whole genome-based phylogenetic tree, strain WK13^T^ showed the highest similarity to *Cardiobacterium valvarum* NCTC 13294^T^ with a dDDH value of 35.7% [GGDC formula 2 (sum gene identities/high-scoring segment pairs (HSPs) length)]. This formula represents the degree of sequence similarity within the homologous regions of the compared genomes. Additionally, GGDC formula 2 is not affected by genome size, making it resilient when working with draft genomes (Fig. 2, Table S1, available in the online Supplementary Material) [30]. Species from the genus *Cardiobacterium* are known to cause endocarditis, and *C. valvarum* was isolated from a patient with endocarditis and ruptured cerebral aneurysm [34]. Interestingly, *C. valvarum* is probably an oral organism but belongs to the *Gammaproteobacteria*, which is phylogenetically quite distinct from the *Betaproteobacteria*. However, WK13^T^ shows only 85.3% 16S rRNA gene sequence similarity in the ‘EMBOSS needle’ PSA with *C. valvarum* but seems to share a higher gene identity in homologous regions than with species that are more closely related in their 16S rRNA gene sequence. The reason for this observation could be horizontal gene transfer, but it needs to be evaluated in further studies. The next closest related strain in the whole genome-based phylogenetic tree is the betaproteobacterium *P. soli* JCM 35595^T^ isolated from paddy soil, with a dDDH value of 19.5% (sum identities/HSP length) [23]. The TYGS 16S rRNA gene sequence-based phylogenetic tree clustered similarly to the tree designed with mega6. *C. valvarum* and *H. influenzae* form the outgroups here (Fig. S1).

The TYGS-calculated G+C content from the three contigs of strain WK13^T^ was 57.94 mol% that is lower than that from the *Propionivibrio* strains, which have between 60.3 and 61.6 mol%.

## Phenotypic characteristics

Characterization of WK13^T^ was carried out with cells grown anaerobically on yeast extract cysteine agar (10% sheep blood) with *C. sputigena* as co-culture. Cell morphology, size and motility were examined using phase-contrast microscopy and scanning electron microscopy (SEM) (JSM-IT100, JEOL). For the SEM analysis, cells were grown on yeast extract cysteine agar (10% sheep blood) with *C. sputigena*. One well of a ten-well hydrophobic-coated diagnostic microscope slide (Erie Scientific Company; Thermo Scientific) was cut off with a glass cutter. Cells were transferred to this well and fixed in 8% formaldehyde for 2–3 days at 4 °C. After dehydration with ethanol, cells were dried by critical point drying (BAL-TEC CPD030) and sputtered (JFC-1200 Fine Coater, JEOL). Cell size was calculated using ImageJ [35]. Gram staining was performed using the method described by Dahouk *et al.* [36]. Oxidase (Bactident^®^ Oxidase, Merck) and catalase assays were carried out as described by Burkhardt and Bauernfeind [37]. Further biochemical characterizations were conducted to determine whether WK13^T^ is capable of utilizing the following substrates: glucose, maltose, lactose, fructose, mannitol and citrate. These experiments were carried out with Diatabs (Rosco, Denmark) as indicated in the manufacturer’s instructions.

**Fig. 3.**
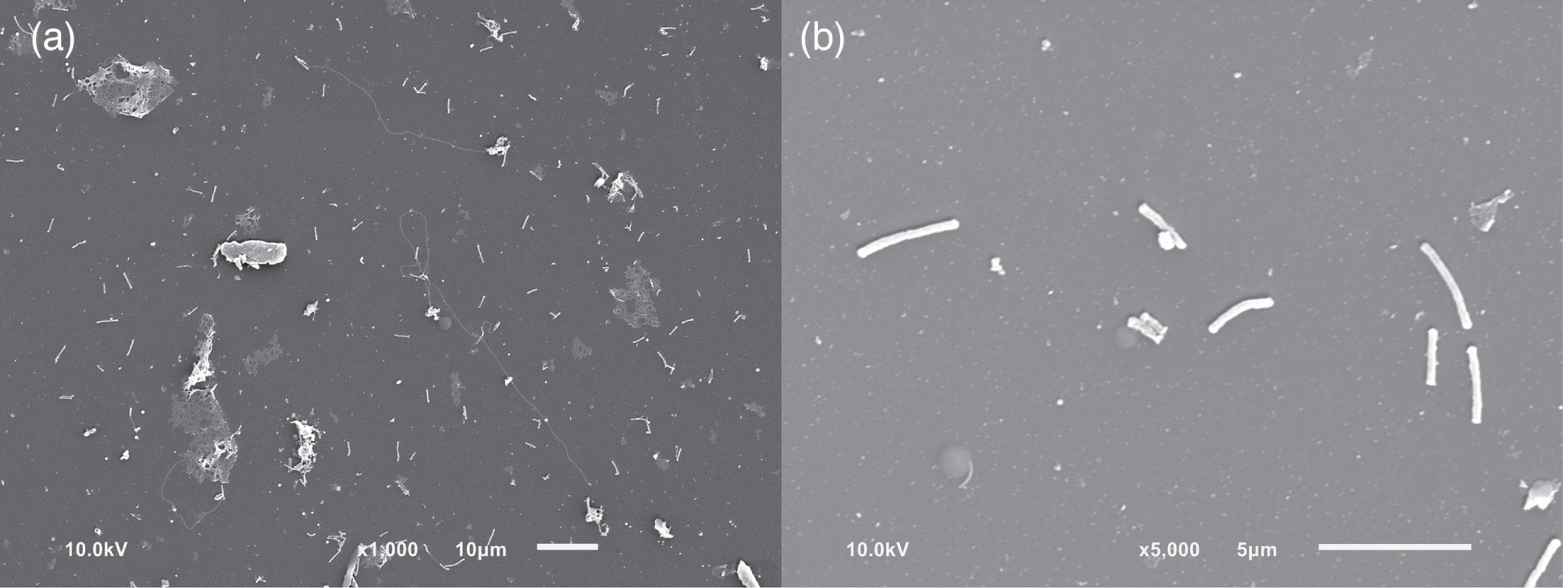
Scanning electron micrographs of cells of *D. hellwigii* WK13^T^. Cells were transferred from yeast extract cysteine agar (10% sheep blood) to diagnostic microscope slides. (a) Rod-shaped cells in 1000-fold magnification. Long filaments can be distinguished. These may represent extrachromosomal DNA. (b) Long, slender rods under 5000-fold magnification.

WK13^T^ formed translucent colonies on yeast extract cysteine agar (10% sheep blood) after 4 days of anoxic incubation (<0.1% O_2_, 15–20% CO_2_) with *C. sputigena* and was Gram-stain-negative. Optimal growth was tested for the temperatures 24 °C, 28 °C, 36 °C and 42 °C and occurred at 36 °C. Cells have been cultured at neutral pH. The cells are slender rods and were between 0.24 and 0.32 µm in width and 1.7–2.4 µm in length. Members of the *Propionivibrio* genus are characterized by the formation of ivory- to white-coloured colonies, and the rods have a wider cell width. Like the *Propionivibrio* species, WK13^T^ cells were motile. Nevertheless, thus far, no flagella could be determined in the SEM for WK13^T^ (Fig. 3). The isolated strain was catalase-positive and oxidase-negative. WK13^T^ was able to utilize glucose, maltose, lactose, fructose and mannitol but not citrate. *P. dicarboxylicus* was not able to utilize glucose, maltose, lactose, fructose and mannitol; *P. pelophilus* was not able to utilize maltose and lactose but citrate; *P. limicola* was not able to utilize any of the tested substrates (maltose was not tested); and *P. soli* was able to utilize glucose and citrate. The biochemical and phenotypic characteristics of the isolate and most closely related taxa are summarized in Table 1.

**Table 1.** Differential characteristics for strain WK13^T^ and most closely related taxa. Strains: 1, *D. hellwigii* gen. nov., sp. nov. WK13^T^ DSM 112713^T^ (data from this study); 2, *P. dicarboxylicus* DSM 5885^T^ [12]; 3, *P. pelophilus* DSM 12018^T^ [11]; 4, *P. limicola* DSM^T^ 6832^T^ [19]; 5, *P. soli* JCM 35595^T^ [23]; 6, *A. restricta* DSM 18626^T^ [18]; 7, *R. gracilis* DSM 110^T^ [20]; 8, *C. valvarum* NCTC 13294^T^ [33]; +, positive; −, negative; nd, no data available

Characteristic	1	2	3	4	5	6	7	8
Catalase	+	−	nd	−	+	+	nd	−
Oxidase	−	nd	nd	−	−	+	nd	+
Gram stain	Negative	Negative	Negative	Negative	Negative	Negative	nd	Negative
Colony pigmentation	Translucent	Ivory	Slimy white	nd	White	Dependent on medium translucent/greenish yellow or cream-coloured	Red to purple violet	Round, opaque, smooth and glistening
Motility	Motile	Motile with a single flagellum	Motile with one polar flagellum	Motile with one polar flagellum	Motile with apical flagella	Motile with one polar flagellum	Motile by polar flagella	−
Morphology	Slender rods	Curved rods	Rods	Slender rods	Rod-shaped	Straight or curved rods	Weakly curved rods	Dependent on medium regular rod/pleomorphic
Size (µm)	0.24–0.32×1.7–2.4	0.5–0.6×1.0–2.0	0.5–0.6×0.9–1.1	0.6–0.7×1.5–2.5	nd	0.5×1.2–2.5	0.3–0.5×1.5–5	1×2–4
Oxygen tolerance	Obligate anaerobic	Obligate anaerobic	Rather oxygen-tolerant anaerobe	Aerotolerant	Facultative anaerobic	Aerobic, capable of microaerophilic growth	Anaerobically in the light	Aerobic, capable of microaerophilic growth
								
Optimum temperature for growth (°C)	36	30	27–30	37	30	37	30	nd
Utilization of:								
Glucose	+	−	+	−	+	−	−	−
Maltose	+	−	−	nd	nd	−	nd	−
Lactose	+	−	−	−	nd	−	nd	−
Fructose	+	−	+	−	nd	−	−	+/−
Mannitol	+	−	+	−	−	nd	−	−
Citrate	−	nd	+	−	+	nd	−	nd
DNA G+C content (mol%)	57.9*	61	60.8	61.6	60.3	67.9	64.5	58.0*
Habitat	Root canal in human oral cavity	Anaerobic mud of freshwater sediments	Anoxic freshwater and estuarine sediments	Anoxic freshwater sediment	Paddy soil	Groundwater	Freshwater lakes and peat bogs	Isolated from human blood; possibly originating from dental plaque

* G+C content calculated with TYGS.

## Chemotaxonomic characterization

Cellular fatty acids were extracted from wet biomass, cultivated on yeast extract cysteine agar supplemented with 10% sheep blood for 8 days, according to the protocol of Christie [38] with slight modifications. Briefly, wet biomass was resuspended in methanol:toluene (1 : 1, v/v) mixed with 50 µl sulphuric acid and incubated overnight at 50 °C. The suspension was mixed with 0.5 ml saturated sodium chloride and extracted twice with hexane:chloroform (4 : 1 v/v). The extracts were evaporated under a stream of nitrogen and reconstituted in *tert-*butyl methyl ether prior to GC analysis. Fatty acid methyl esters were analysed via GC coupled to a flame ionization detector using the Sherlock Microbial Identification System (MIDI, version 6.1, TSBA6 database). The identity of all fatty acids was validated by GC-MS as described earlier [39]. Double bond positions were determined after derivatization to dimethyl disulphide species and subsequent GC-MS analysis [40].

Respiratory quinones were extracted from wet biomass and analysed by HPLC coupled to a diode array detector and a high-resolution mass spectrometer [39,41].

Polar lipids were extracted from wet biomass using a modified Bligh and Dyer extraction [42]. Briefly, samples were extracted twice with methanol:dichloromethane (DCM):0.3% NaCl (2 : 1 : 0.8, v/v/v) by ultrasonication at ambient temperature for 10 min. The DCM phases were combined and subsequently extracted twice with 0.3% NaCl. The combined DCM extracts were evaporated under a stream of nitrogen, reconstituted in hexane/isopropanol/water (718 : 271 : 10 v/v/v) and filtered through a regenerated cellulose syringe filter (Minisart RC4, Sartorius). Intact polar lipids were analysed by HPLC-MS as described previously [43].

The predominant cellular fatty acids of strain WK13^T^ (≥ 5 %) are C_16 : 0_, C_18 : 0_, C_16 : 1_* ω*7c, C_18 : 1_* ω*9c and C_18 : 2_* ω*6,9c (Table S2). The fatty acid profile of *P. soli* is different in terms of the absence of C_18 : 0_ and C_18 : 2_* ω*6,9c [23]. However, the comparability is limited due to the differences in lifestyle, e.g. different temperature optima and medium requirements.

The polar lipid profile of strain WK13^T^ consists of diphosphatidylglycerol (DPG), phosphatidylethanolamine (PE), phosphatidylserine (PS), phosphatidylcholine (PC) and lysophosphatidylcholine (LPC). This lipid profile is partially different from that of *P. soli* for which PS, PE, phosphatidylglycerol and DPG were predicted based on genome annotations [23].

Respiratory quinones were not detected, which differs considerably from the closest relative, *P. soli*, which was shown to contain ubiquinone-7 [23]. In contrast to the *Propionivibrio* strains, strain WK13^T^ prefers to grow in co-culture with *Capnocytophaga* species. We harvested WK13^T^ biomass from a plate with this co-culture. However, menaquinone-6 as the typical quinone of *C. sputigena* was not detectable [44].

On the basis of ecologic, phylogenetic, phenotypic and chemotaxonomic analysis, strain WK13^T^ should be classified as a member of a novel species in a new genus, for which the name *D. hellwigii* gen. nov., sp. nov. is proposed.

## Description of *Dentiradicibacter* gen. nov.

*Dentiradicibacter* (Den.ti.ra.di.ci.bac'ter. L. masc. n. *dens*, tooth; L. fem. n. *radix*, root; N.L. masc. n. *bacter*, a rod; N.L. masc. n. *Dentiradicibacter*, a rod from the root of a tooth).

Cells are Gram-stain-negative, anaerobic rods that are motile. Cells were isolated from human samples and grow on blood agar plates in an anoxic atmosphere (<0.1% O_2_, 15%–20% CO_2_). Good growth occurs only in co-culture with, e.g. *C. sputigena*. The major fatty acids (≥ 5.0 %) are C_16 : 0_, C_18 : 0_, C_16 : 1_* ω*7c, C_18 : 1_* ω*9c and C_18 : 2_* ω*6,9c. The major polar lipids are DPG, PE, PS, PC and LPC. Respiratory quinones are not detected. The type species is *D. hellwigii*.

## Description of *Dentiradicibacter hellwigii* sp. nov.

*Dentiradicibacter hellwigii* (hell.wi.gii.i N.L. gen. masc. n. *hellwigii* of Hellwig, named in honour of Elmar Hellwig, recently retired Professor for Operative Dentistry and Periodontology, who was strongly committed to the establishment of oral microbiology in Freiburg, Germany).

In addition to the characteristics mentioned in the description of the genus, translucent colonies are observed on yeast extract cysteine agar with sheep blood after 4 days. Cells are slender rods and 0.24–0.32 µm in width and 1.7–2.4 µm long. Optimal growth occurred at 36 °C. Cells were catalase-positive and oxidase-negative and able to utilize glucose, maltose, lactose, fructose and mannitol but not citrate.

The type strain, WK13^T^ (=DSM 112713^T^=NCTC 14938^T^), was isolated from the root canal of a human patient. The draft genome sequence of strain WK13^T^ is available at DDBJ/ENA/GenBank under accession number JBEUWX000000000. The 16S rRNA gene sequence of strain WK13^T^ has the GenBank accession number OQ442336.

## supplementary material

10.1099/ijsem.0.006690 Uncited Supplementary Material 1.

## Abbreviations

BGP: basal glucose phosphate
DCM: dichlormethane
dDDH: digital DNA–DNA hybridization
DPG: diphosphatidylglycerol
GBDP: Genome blast Distance Phylogeny
GGDC: genome-to-genome distance calculator
HSPs: high-scoring segment pairs
LPC: lysophosphatidylcholine
ML: maximum likelihood
NJ: neighbour-joining
PC: phosphatidylcholine
PE: phosphatidylethanolamine
PS: phosphatidylserine
PSA: pairwise sequence alignment
SEM: scanning electron microscopy
TYGS: Type (Strain) Genome Server
