# Supplementary material for: Dentiradicibacter hellwigii gen. nov., sp. nov., isolated from a secondary infected root canal in the human oral cavity
Source: Int J Syst Evol Microbiol. 2025 Mar 5;75(3):006690. doi: 10.1099/ijsem.0.006690 (PMC11881992; doi:10.1099/ijsem.0.006690)
Supplement: Uncited Supplementary Material 1. [file ijsem-75-06690-s001.pdf]

Dentiradicibacter hellwigii gen. nov., sp nov., isolated from a secondary infected root canal in the human oral cavity.

Sibylle Bartsch, Annette Wittmer, Ann-Kathrin Weber, Meina Neumann-Schaal, Jacqueline Wolf, Sabine Gronow, Jake David Turnbull, Christian Tennert, Georg Häcker, Fabian Cieplik, Ali Al-Ahmad

Supplementary figures and tables

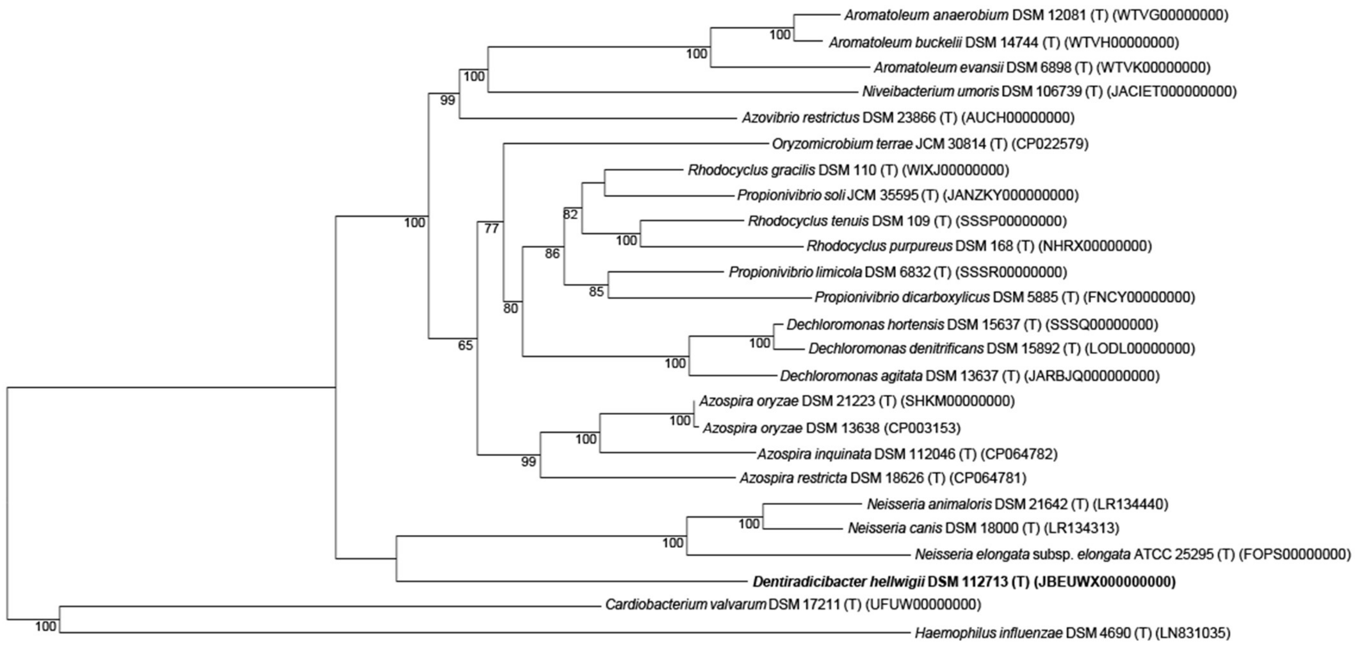

Figure S1. 16S rDNA sequence Genome BLAST Distance Phylogeny (GBDP) tree. Tree inferred with FastME 2.1.6.1 from GBDP distances calculated from 16S rRNA gene sequences (1). The branch lengths are scaled in terms of GBDP distance formula d5. The numbers below branches are GBDP pseudo-bootstrap support values > 60 % from 100 replications, with an average branch support of 89.3 %. The tree was rooted at the midpoint (2).

Table S1: **Pairwise comparison of digital DNA-DNA hybridization (dDDH) values between WK13^T and selected type-strain genomes.** The dDDH values are provided along with their confidence intervals (C.I.) for the three different GBDP formulas: formula d0 (a.k.a. genome to genome distance calculator (GGDC) formula 1): length of all high-scoring segment pairs (HSPs) divided by total genome length; formula d4 (a.k.a. GGDC formula 2): sum of all identities found in HSPs divided by overall HSP length; formula d6 (a.k.a. GGDC formula 3): sum of all identities found in HSPs divided by total genome length. Formulas d0 and d6 measure the similarity or dissimilarity of the compared genomes based on their gene content. In contrast, formula d4 quantifies the degree of sequence identity within the homologous regions of these genomes. Importantly, d4 is not influenced by genome length, making it reliable even when working with draft genomes (3). dDDH - digital DNA-DNA hybridization; C.I. - confidence intervals.

Type Strain Genome Server								
**Query strain**	**Subject strain**	**dDDH (d0, in %)**	**C.I. (d0, in %)**	**dDDH (d4, in %)**	**C.I. (d4, in %)**	**dDDH (d6, in %)**	**C.I. (d6, in %)**	**G+C content difference (in %)**
D. hellwigii DSM 112713 (T) (JBEUWX000000000)	*Cardiobacterium valvarum* DSM 17211 (T) (UFUW00000000)	12,9	[10.2 - 16.2]	35,7	[33.3 - 38.2]	13,4	[11.0 - 16.1]	0,05
D. hellwigii DSM 112713 (T) (JBEUWX000000000)	*Neisseria animaloris* DSM 21642 (T) (LR134440)	12,6	[10.0 - 15.9]	27,3	[24.9 - 29.7]	13	[10.7 - 15.8]	8,65
D. hellwigii DSM 112713 (T) (JBEUWX000000000)	*Neisseria canis* DSM 18000 (T) (LR134313)	12,6	[10.0 - 15.9]	26	[23.6 - 28.4]	13	[10.7 - 15.8]	8,2
D. hellwigii DSM 112713 (T) (JBEUWX000000000)	*Haemophilus influenzae* DSM 4690 (T) (LN831035)	12,6	[9.9 - 15.9]	24,8	[22.4 - 27.2]	13	[10.7 - 15.8]	19,71
D. hellwigii DSM 112713 (T) (JBEUWX000000000)	*Aromatoleum evansii* DSM 6898 (T) (WTVK00000000)	12,6	[9.9 - 15.9]	21,1	[18.8 - 23.5]	13	[10.7 - 15.8]	8,04
D. hellwigii DSM 112713 (T) (JBEUWX000000000)	*Aromatoleum buckelii* DSM 14744 (T) (WTVH00000000)	12,8	[10.1 - 16.0]	20,5	[18.3 - 22.9]	13,1	[10.8 - 15.9]	7,39
D. hellwigii DSM 112713 (T) (JBEUWX000000000)	*Dechloromonas denitrificans* DSM 15892 (T) (LODL00000000	12,8	[10.1 - 16.1]	19,8	[17.6 - 22.2]	13,2	[10.9 - 16.0]	3,81
D. hellwigii DSM 112713 (T) (JBEUWX000000000)	*Aromatoleum anaerobium* DSM 12081 (T)(WTVG00000000)	12,6	[10.0 - 15.9]	19,8	[17.6 - 22.2]	13	[10.7 - 15.8]	7,77
D. hellwigii DSM 112713 (T) (JBEUWX000000000)	*Rhodocyclus tenuis* DSM 109 (T) (SSSP00000000)	13	[10.3 - 16.3]	19,8	[17.6 - 22.3]	13,3	[11.0 - 16.1]	6,77
D. hellwigii DSM 112713 (T) (JBEUWX000000000)	*Propionivibrio limicola* DSM 6832 (T) (SSSR00000000)	13,3	[10.6 - 16.7]	19,6	[17.4 - 22.0]	13,7	[11.3 - 16.4]	2,38
D. hellwigii DSM 112713 (T) (JBEUWX000000000)	*Rhodocyclus purpureus* DSM 168 (T) (NHRX00000000)	12,9	[10.2 - 16.2]	19,6	[17.4 - 22.0]	13,3	[10.9 - 16.1]	8,2
D. hellwigii DSM 112713 (T) (JBEUWX000000000)	*Rhodocyclus gracilis* DSM 110 (T) (WIXJ00000000)	13	[10.3 - 16.3]	19,3	[17.1 - 21.7]	13,4	[11.0 - 16.2]	6,57
D. hellwigii DSM 112713 (T) (JBEUWX000000000)	Dechloromonas hortensis DSM 15637 (T) (SSSQ00000000)	12,8	[10.1 - 16.1]	19,1	[16.9 - 21.5]	13,2	[10.8 - 15.9]	3,5
D. hellwigii DSM 112713 (T) (JBEUWX000000000)	Azospira oryzae DSM 21223 (T) (SHKM00000000)	12,8	[10.1 - 16.0]	18,7	[16.6 - 21.1]	13,1	[10.8 - 15.9]	8,37

Table S2: Fatty acid and polar lipid composition of strain WK13^{T}. Cells have been cultivated on yeast extract cysteine agar with 10 % sheep blood at 37 °C. tr – traces (fatty acid abundance < 1 %). DPG – diphosphatidylglycerol, PE – phosphatidylethanolamine, PS – phosphatidylserine, PC – phosphatidylcholine, LPC – lysophosphatidylcholine.

**Fatty acid**	**Abundance [%]**
$C_{13:0}$	tr
$C_{12:0}$ 3OH	1.3
$C_{14:0}$	3.9
$C_{16:0}$ iso	tr
$C_{16:1}$ ω9c	tr
$C_{16:1}$ ω7c	10.6
$C_{16:0}$	42.6
$C_{17:0}$ iso	tr
$C_{17:0}$ anteiso	1.0
$C_{17:0}$	3.7
$C_{18:2}$ ω6,9c	5.0
$C_{18:1}$ ω9c	10.0
$C_{18:1}$ ω7c	2.7
$C_{18:0}$	15.9
**Polar lipids**	DPG, PE, PS, PC, LPC

1. Lefort, V., R. Desper & O. Gascuel. (2015) FastME 2.0: A Comprehensive, Accurate, and Fast Distance-Based Phylogeny Inference Program. *Mol Biol Evol* **32**: 2798-2800. https://doi.org/10.1093/molbev/msv150
2. Farris, J.S. (1972) Estimating Phylogenetic Trees from Distance Matrices. *American Naturalist* **106**: 645-668.
3. Meier-Kolthoff, J.P., A.F. Auch, H.-P. Klenk & M. Göker. (2013) Genome sequence-based species delimitation with confidence intervals and improved distance functions. *BMC Bioinformatics* **14**: 60. https://doi.org/10.1186/1471-2105-14-60
